# Supplementary material for: The O-GlcNAc Transferase Intellectual Disability Mutation L254F Distorts the TPR Helix
Source: Cell Chem Biol. 2018 May 17;25(5):513–518.e4. doi: 10.1016/j.chembiol.2018.03.004 (PMC5967971; doi:10.1016/j.chembiol.2018.03.004)
Supplement: Document S1. Figures S1–S3 [file mmc1.pdf]

Cell Chemical Biology, Volume *25*

Supplemental Information

The *O*-GlcNAc Transferase Intellectual Disability Mutation L254F Distorts the TPR Helix

Mehmet Gundogdu, Salomé Llabrés, Andrii Gorelik, Andrew T. Ferenbach, Ulrich Zachariae, and Daan M.F. van Aalten

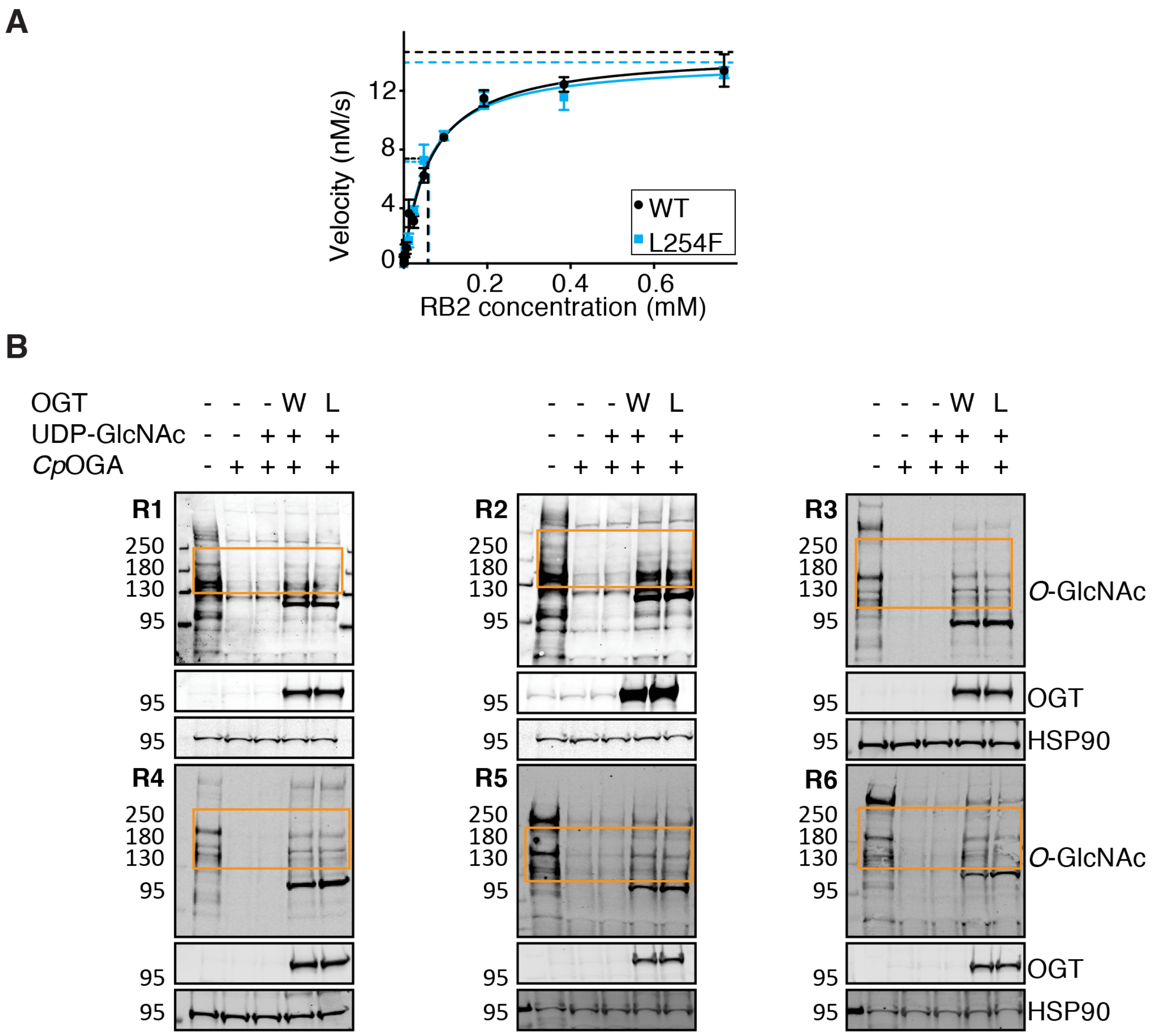

Supplemental figure 1: *in vitro* characterisation of OGT$_{L254F}$. Related to Figure 1B

(A) Michaelis-Menten kinetics of OGT wild type and mutant against RB2 peptide. X- and y-axis intercepts of the dashed lines mark the Michaelis constant (K_M) and maximum velocity (V_{max}) of the reaction. Error bars represent standard deviation of three replicates. **(B)** Western blot analyses showing OGT activity against de-*O*-GlcNAcylated HEK-293 cell lysate. Results represent six biological replicates and orange boxes depict the area that was used for quantification. *Cp*OGA: *Clostridium perfringens*; L: L254F; W: wild type.

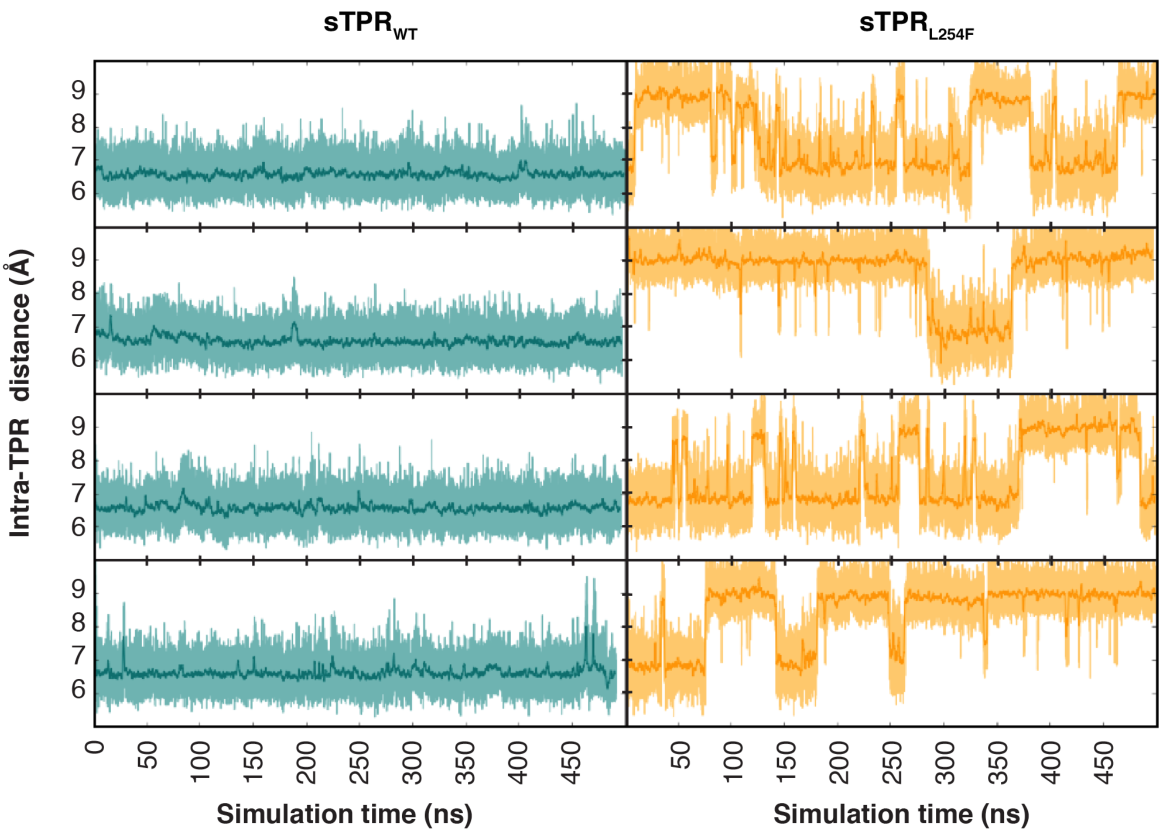

Supplemental figure 2: Analysis of the intra-TPR repeat distance. Related to Figures 2B and 2C

Molecular dynamics simulation data investigating the intra TPR distance observed in sTPR$_{WT}$ and sTPR$_{L254F}$ are shown on left and right panels, respectively. sTPR: simplified TPR; TPR: tetratricopeptide repeat.

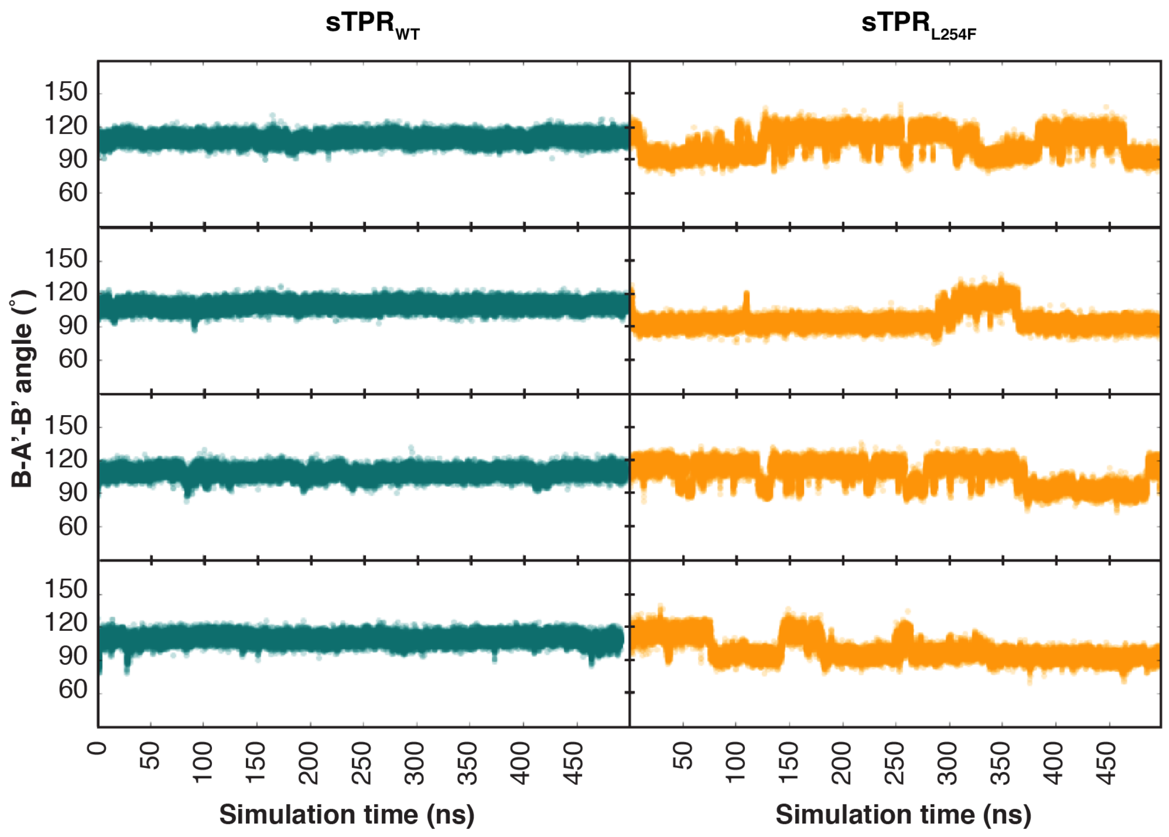

Supplemental figure 3: Analysis of the angle B-A'-B'. Related to Figures 2B and 2C

Molecular dynamics simulation data investigating the B-A'-B' angle observed in sTPR$_{WT}$ and sTPR$_{L254F}$ are shown on left and right panels, respectively. sTPR: simplified TPR; TPR: tetratricopeptide repeat.
