# Supplementary material for: The O-GlcNAc Transferase Intellectual Disability Mutation L254F Distorts the TPR Helix
Source: Cell Chem Biol. 2018 May 17;25(5):513–518.e4. doi: 10.1016/j.chembiol.2018.03.004 (PMC5967971; doi:10.1016/j.chembiol.2018.03.004)
Supplement: Document S2. Article plus Supplemental Information [file mmc2.pdf]

Brief Communication

Cell Chemical Biology

The *O*-GlcNAc Transferase Intellectual Disability Mutation L254F Distorts the TPR Helix

Graphical Abstract

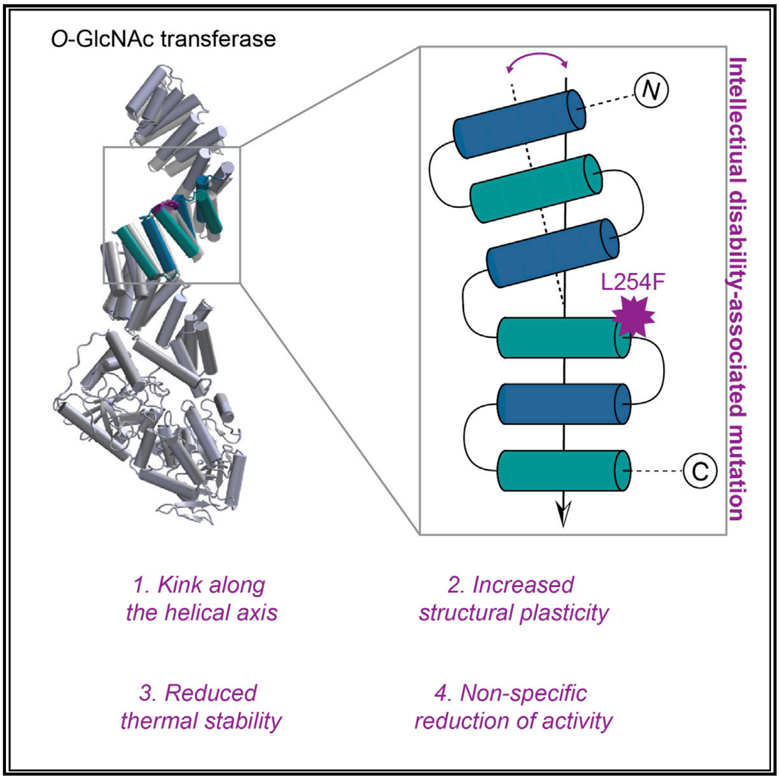

Highlights

- The intellectual disability L254F mutation in OGT affects activity
- The L254F mutation leads to shifts up to 12 Å in the OGT structure
- Thermal denaturing studies reveal reduction in TPR stability caused by L254F
- Simulations suggest the presence of alternate TPR_{L254F} conformations

Authors

Mehmet Gundogdu, Salomé Llabrés, Andrii Gorelik, Andrew T. Ferenbach, Ulrich Zachariae, Daan M.F. van Aalten

Correspondence

dmfvanaalten@dundee.ac.uk

In Brief

Gundogdu et al. demonstrate how the intellectual disability-associated mutation in *O*-GlcNAc transferase (OGT), L254F, leads not only to large shifts in OGT structure and altered dynamics in solution, but also a non-specific reduction in activity, providing the first molecular characterization of such a mutation.

Gundogdu et al., 2018, Cell Chemical Biology *25*, 513–518
May 17, 2018 © 2018 The Authors. Published by Elsevier Ltd.
https://doi.org/10.1016/j.chembiol.2018.03.004

CellPress

The *O*-GlcNAc Transferase Intellectual Disability Mutation L254F Distorts the TPR Helix

Mehmet Gundogdu,[1] Salomé Llabrés,[2] Andrii Gorelik,[1] Andrew T. Ferenbach,[1] Ulrich Zachariae,[2,3] and Daan M.F. van Aalten[1,4,*]
[1]Centre for Gene Regulation and Expression, School of Life Sciences, University of Dundee, Dundee DD1 5HE, UK
[2]Division of Computational Biology, School of Life Sciences, University of Dundee, Dundee DD1 5HE, UK
[3]School of Physics and Engineering, University of Dundee, Dundee DD1 5HE, UK
[4]Lead Contact
*Correspondence: dmfvanaalten@dundee.ac.uk
https://doi.org/10.1016/j.chembiol.2018.03.004

SUMMARY

***O*-linked β-*N*-acetyl-D-glucosamine (*O*-GlcNAc) transferase (OGT) regulates protein *O*-GlcNAcylation, an essential post-translational modification that is abundant in the brain. Recently, *OGT* mutations have been associated with intellectual disability, although it is not understood how they affect OGT structure and function. Using a multidisciplinary approach we show that the L254F OGT mutation leads to conformational changes of the tetratricopeptide repeats and reduced activity, revealing the molecular mechanisms contributing to pathogenesis.**

INTRODUCTION

O-linked β-*N*-acetyl-D-glucosamine (*O*-GlcNAc) transferase (OGT) and hydrolase (OGA) control a dynamic, reversible, and tightly regulated post-translational modification termed *O*-GlcNAcylation (Yang and Qian, 2017). OGT is abundantly expressed in the brain (Okuyama and Marshall, 2003) and localizes to synaptosomes (Cole and Hart, 2001). Accordingly, OGT has been linked to regulation of axonal and dendritic morphology (Francisco et al., 2009), axonal transport of mitochondria (Pekkurnaz et al., 2014) and, at the behavioral level, to response and habituation to environmental stimuli (Timbers et al., 2017). These findings implicate *O*-GlcNAcylation in various brain functions, including learning and memory.

Intellectual disability (ID) is a disease defined by early-onset impairment of cognitive function and limitation of adaptive behavior (Ropers, 2010). The most common causes of ID are monogenic mutations in over 650 genes (Kochinke et al., 2016). Recently, mutations in the N terminus of human *OGT* have been associated with ID, namely, A319T, L254F, R284P, and Δ155-177 (Bouazzi et al., 2015; Niranjan et al., 2015; Willems et al., 2017) (Figure 1A). Two recent studies have reported that while some of these mutations affect OGT activity *in vitro*, *O*-GlcNAc homeostasis appears to be maintained in patient-derived cells by reduced *OGA* expression (Vaidyanathan et al., 2017; Willems et al., 2017). It is as yet unclear how these ID-associated mutations affect OGT structure and function and result in the ID phenotype.

OGT possesses 13.5 tetratricopeptide repeats (TPRs) at the N terminus, which harbor all currently known ID-associated mutations (Lubas et al., 1997) (Figure 1A). TPR-containing proteins are ubiquitous and functionally versatile (Zeytuni and Zarivach, 2012). TPRs were first described in protein phosphatase 5 (PP5), where they negatively regulate catalytic activity in a ligand-dependent manner (Connarn et al., 2014). In OGT, the TPRs form a 120-Å superhelix, which serves as a potential interaction surface for substrates and binding partners (Jínek et al., 2004; Zeytuni and Zarivach, 2012). Two modes of interaction have been proposed. The central channel generated by the TPR superhelix has been shown to engage some substrates through an asparagine ladder, which interacts with the carbonyl and amide groups of the substrate backbone in a sequence-independent fashion (Lazarus et al., 2013; Rafie et al., 2017). In addition, the superhelical grooves on the TPR superhelix may interact with substrates, with different TPRs recruiting distinct target proteins in a sequence-specific fashion, in a manner similar to HSP70/90 organizing protein (Scheufler et al., 2000). These two models explain how OGT can modify thousands of intracellular proteins on specific sites while also serving as a scaffold in multi-protein complexes. Here, we demonstrate that the L254F mutation in OGT induces folding defects in the TPR superhelix, suggesting that changes in interactions with substrates and/or binding partners may underpin the ID disease phenotype.

RESULTS AND DISCUSSION

The ID-associated mutation L254F (Vaidyanathan et al., 2017) is located on TPR helix 7, distant from the active site (Figure 1A). *In vitro* OGT activity was tested with a short acceptor peptide derived from the RB2 protein (Pathak et al., 2015). In this assay, the L254F mutation had no effect on steady-state kinetics of OGT (peptide K_M = 0.6 mM, V_{max} = 15 nM/s for both enzymes; Figure S1A). Next, we measured OGT activity against de-*O*-GlcNAcylated HEK-293 cell lysate containing a multitude of substrates for OGT, some of which may be recognized by the superhelical grooves found on OGT TPRs. We observed that addition of recombinant OGT_{L254F} was not able to fully restore the amount of *O*-GlcNAc transfer to that of the OGT_{WT}-treated

© 2018 The Authors. Published by Elsevier Ltd.
This is an open access article under the CC BY license (http://creativecommons.org/licenses/by/4.0/).

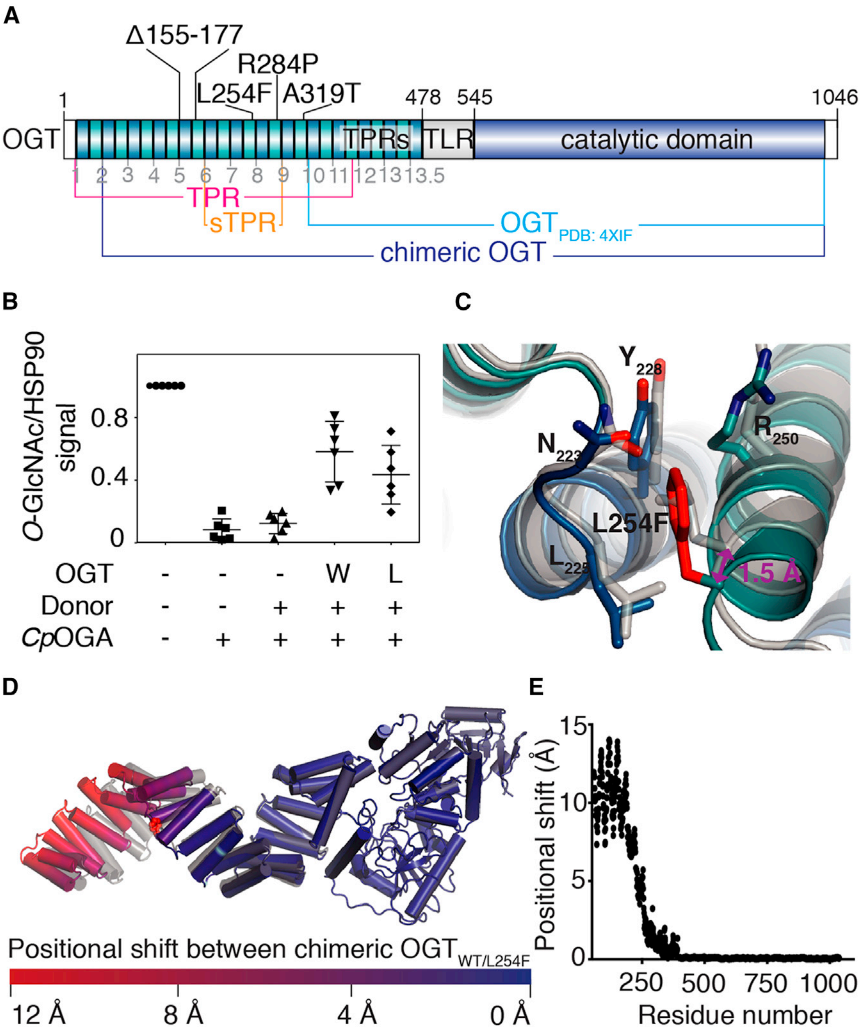

Figure 1. *In Vitro* Characterization of OGT_{L254F}

(A) Schematic representation of OGT highlighting the intellectual disability-associated mutations and all the constructs used in this study.

(B) Scatterplot showing OGT activity against deglycosylated HEK-293 cell lysate, with the data averaged from six replicates and the error bars showing SD. See also Figure S1B.

(C) Superposition of the $TPR_{WT/L254F}$ crystal structures at the site of mutation. The gray and colored cartoons are that of TPR_{WT} (PDB: 1W3B; Jínek et al., 2004) and TPR_{L254F} (PDB: 6EOU) structures, respectively.

(D) Overlay of the chimeric $OGT_{WT/L254F}$ structures. The wild-type structure is colored gray, while the mutant structure is colored to reflect the positional shift of each C_α atom between the two structures.

(E) Graph showing the positional shift between equivalent C_α atoms between chimeric OGT_{WT} (PDB: 4XIF [Pathak et al., 2015] and PDB: 1W3B [Jínek et al., 2004]) and OGT_{L254F} (PDB: 4XIF [Pathak et al., 2015] and PDB: 6EOU) as a function of residue number.

L, L254F; OGT, O-GlcNAc transferase; sTPR, simplified TPR; TPR, tetratricopeptide repeat; W, wild-type.

control (Figures 1B and S1B). Thus, in our hands the ID OGT L254F mutation shows effects on *in vitro* activity.

A single TPR motif consists of an anti-parallel pair of α helices, named helix A and B, which are held together by interactions between conserved residues in the 34-amino-acid consensus sequence W_4-L_7-G_8-Y_{11}-A_{20}-F_{24}-A_{27}-P_{32} (Jínek et al., 2004; Lamb et al., 1995). The W_4-L_7-G_8-Y_{11} motif on helix A forms a hydrophobic pocket into which the bulky residue of the A_{20}-F_{24}-A_{27} motif on helix B is lodged. The OGT TPRs possess two additional features: helix A contains a ladder of conserved asparagines (N_6) on TPRs 2–13.5; helix B contains a series of large aliphatic residues on TPRs 6–13.5 (Ψ_{30}; where Ψ represents Leu, Ile, or Val). While the N_6 ladder is involved in substrate recognition (Jínek et al., 2004; Lazarus et al., 2013; Rafie et al., 2017), the series of large aliphatic residues (Ψ_{30}), of which Leu254 is part, interdigitate with aliphatic residues found at the first position within the TPR motifs (X_1; where X represents Leu, Ala, or Pro). To investigate structural changes attributable to the L254F mutation, we determined the crystal structure of the mutant TPR domain (TPR_{L254F}). Recombinant protein was obtained from *Escherichia coli* using the construct boundaries previously employed to crystallize the wild-type OGT TPR domain (TPR_{WT}, Figure 1A) (Jínek et al., 2004). Diffraction data were collected to 1.75 Å (Table 1) and initial refinement starting from the TPR_{WT} structure required substantial rebuilding of the terminal TPRs, an early indication of considerable conformational changes. Indeed, the overall root-mean-square deviation (RMSD) on 343 C_α atoms of the refined TPR_{L254F} structure versus that of TPR_{WT} was 1.6 Å.

In wild-type OGT, L_{254} occupies the interface between the helices of TPR7, with its side chain constricted in a pocket formed by the surrounding residues N_{223}, L_{225}, Y_{228}, and R_{250} (Figure 1C). Mutation of L_{254} to the bulkier Phe appears to be accommodated in this pocket, however, by causing small changes in torsion angles of the residues lining the pocket and by displacing TPR helix 7B away from helix 7A by 1.5 Å (Figure 1C). We examined the effects of this change in the context of the full-length protein, modeled by merging the $TPR_{WT/L254F}$ structures and an OGT structure containing the catalytic domain (residues 325–1,038; PDB: 4XIF [Pathak et al., 2015]) using the overlapping TPRs (residues 325–381) as superposition anchor (Figures 1A and 1D). This reveals a deviation from the wild-type TPR geometry that is propagated toward the N terminus (Figures 2D and 2E). Increasing shifts of the TPRs starting from the mutation site lead to a maximum shift of 12 Å for the N-terminal TPR (Figures 2D and 2E). Thus, the L254F mutation causes a distortion of the TPR helix.

To investigate possible effects of the L254F mutation on stability of the TPRs, we determined the melting temperatures of TPR_{WT} and TPR_{L254F} using differential scanning fluorimetry. A monophasic, sigmoidal melting curve was obtained for TPR_{WT}

Table 1. Scaling and Model-Building Statistics of the TPR_{L254F} Crystal Structure

	TPR_{L254F}
Data Collection	
Space group	$C222_1$
Cell dimensions	
α, β, γ (°)	44.00, 203.16, 116.87
a, b, c (Å)	90.00, 90.00, 90.00
Resolution (Å)	46.58–1.75 (1.75–1.78)
R_{sym} or R_{merge}	0.05 (0.57)
$I/\sigma I$	13.00 (2.00)
Completeness (%)	99.70 (99.80)
Redundancy	4.1 (4.1)
Refinement	
Resolution (Å)	46.58–1.75 (1.75–1.78)
No. of reflections	54,404 (3,607)
R_{work}/R_{free}	0.19/0.23
No. of atoms	3,002
Protein	2,722
Ligand/ion	NA
Water	280
B factors	
Protein	38.40
Ligand/ion	NA
Water	42.52
RMSDs	
Bond lengths (Å)	0.02
Bond angles (°)	2.00

Related to Figures 1C and 1D. Values in parentheses represent the highest-resolution shell.

with an inflection point (T_m) of 58°C (Figure 2A). The melting curve for TPR_{L254F} was shifted, yielding a T_m of 55°C, indicative of reduced thermal stability (Figure 2A). This is in agreement with previous characterization of OGT_{L254F} in patient-derived cells, where the L254F mutation was found to reduce OGT half-life by 2-fold (Vaidyanathan et al., 2017). Thus it appears that the L254F mutation destabilizes OGT.

To explore the mechanisms underlying the reduction in thermal stability, we performed atomistic molecular dynamics simulations (2 μs) using a fragment of the $TPR_{WT/L254F}$ crystal structures, comprising TPRs 6, 7, and 8 (residues 189–294; hereafter $sTPR_{WT}$ and $sTPR_{L254F}$, respectively; Figure 1A). In these simulations we monitored the intra-TPR distance between the C_α atoms of L_{225} (the first residue of helix 7A) and L_{254} (the last residue of helix 7B), the TPR6-7 B-A′-B′ angle formed between the C_α atoms of residues L_{220}, L_{225}, and L_{254}, and the χ_1 dihedral angle of residue 254 (Figure 2B). The intra-TPR distance and the angle B-A′-B′ were stable in the $sTPR_{WT}$ simulations (Figures 2C, S2, and S3), while in the $sTPR_{L254F}$ simulations higher conformational plasticity was observed, showing a partition between two major conformations (Figures 2B and 2C). In $sTPR_{L254F}$ conformation 1 ($sTPR_{L254F\text{-}C1}$), the F_{254} side chain occupies the same position as observed in the TPR_{L254F} crystal structure, while in $sTPR_{L254F}$ conformation 2 ($sTPR_{L254F\text{-}C2}$), the F_{254} phenyl moiety interacts with the side chains of N_{223}, L_{225}, and Y_{228} and the backbone of the F_{224} and R_{250} (Figure 2B). In $sTPR_{L254F\text{-}C2}$ the F_{254} side chain adopts a different conformation, with its aromatic ring positioned parallel to the N_{223} backbone amide in a manner that enables a phenyl-amide interaction. This is accompanied by a 140° ± 17° shift in the F_{254} χ_1 dihedral angle, which in turn distorts the TPR geometry, increasing the intra-TPR distance by 2.1 ± 0.1 Å and shifting the B-A′-B′ angle by approximately 21° ± 7° (Figure 2B). Similar to the conformational rearrangement described for the TPR_{L254F} crystal structure, these local changes propagate through subsequent TPR repeats and modify the overall geometry of the protein. Thus, the L254F mutation destabilizes the interface between TPRs 6 and 7.

The data presented here show that the L254F mutation causes a subtle structural distortion at the mutation site that propagates through the TPR superhelix, resulting in a substantial displacement of the N-terminal TPRs and a markedly increased structural plasticity compared with the TPR_{WT}. Although *in vitro* assays show global effects on the *O*-GlcNAc proteome, they are modest. However, given that *OGT* is essential for life from stem cells to vertebrates and resides on the X chromosome, it is likely that only relatively subtle mutations are tolerated in males. Moreover, it is possible that the ID-associated mutations result in misrepresentation of a distinct subset of the *O*-GlcNAc proteome in different cell lineages, or under certain stimuli. In light of this, it is interesting to note that all OGT ID mutations reported to date are at some distance from the active site (Figure 1A). In addition to effects on the *O*-GlcNAc proteome, it is possible that the conformational changes we observe in the TPRs affect the OGT interactome. For example, mSin3A (Yang et al., 2002), TRAK1 (Iyer and Hart, 2003), TET2, and TET3 (Deplus et al., 2013) all rely on the six N-terminal TPRs for their recruitment onto OGT, while Atx-10 recruitment on OGT is mediated by TPRs 6–8 (März et al., 2006). The work described here forms a platform for the future dissection of these different roles of OGT.

SIGNIFICANCE

***O*-GlcNAc was discovered more than three decades ago, and more than 1,000 proteins in the human proteome are known to be *O*-GlcNAc modified. However, there is still a substantial gap in our understanding of how *O*-GlcNAcylation regulates protein function and downstream cellular pathways. An inroad into this became possible with the recent discovery that patients with mutations in OGT suffer from ID, the clearest evidence yet linking *O*-GlcNAc to neuronal function, in addition to previous reports implying dysregulation of *O*-GlcNAc in neurodegeneration. Although several OGT mutations linked to ID have recently been reported, it is not understood how these mutations affect OGT at the molecular level. This article is the first to describe the substantial molecular consequences of such a mutation. The L254F mutation resides in the TPR helix, which facilitates OGT substrate recognition. Using X-ray crystallography we have uncovered that this mutation leads to shifts**

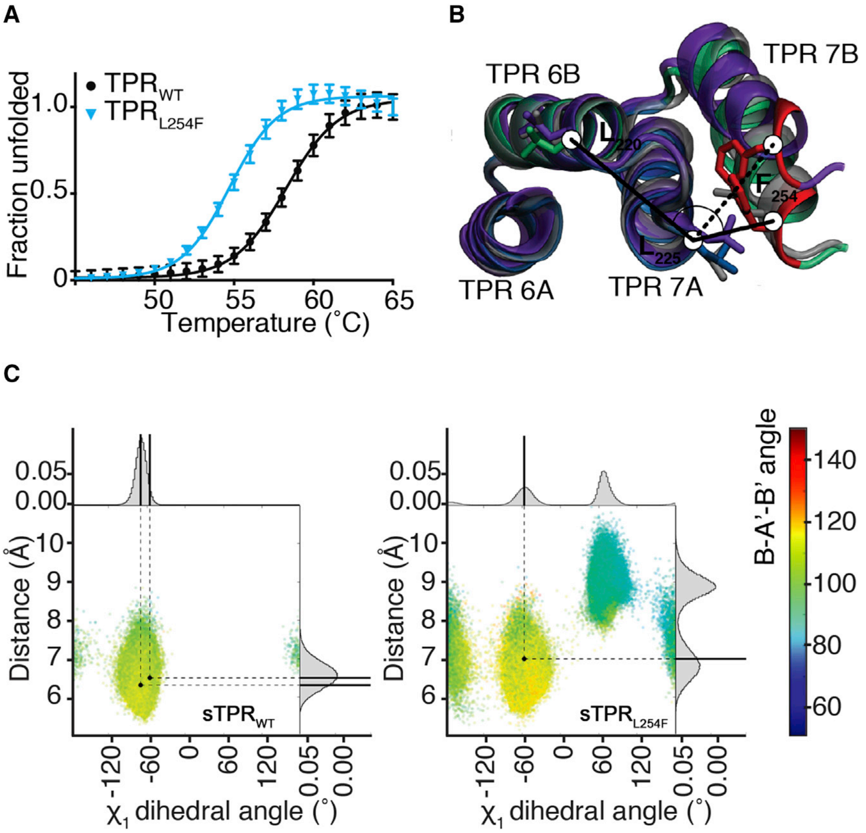

Figure 2. Characterization of the Effects of the ID-Associated Mutation on OGT TPR Stability and Dynamics

(A) Thermal denaturing curve showing fraction of unfolded TPR_{WT} and TPR_{L254F} constructs as a function of temperature. Data averaged from seven replicates were fitted to a Boltzmann sigmoidal curve equation, with error bars representing SD.

(B) Superposition of $sTPR_{WT}$ (gray), $sTPR_{L254F\text{-}C1}$ (green), and $sTPR_{L254F\text{-}C2}$ (purple), with the B-A′-B′ angle and intra-TPR distance demarcated with solid and dashed lines, respectively. See also Figures S2 and S3.

(C) Graphs of $sTPR_{WT}$ (left) and $sTPR_{L254F}$ (right) conformational populations in the molecular dynamics simulations, with the χ_1 dihedral angle of residue 254 shown on the x axis, the intra-TPR repeat distance shown on the y axis, and the angle B-A′-B′ shown as a color scale. The B-A′-B′ values observed in the crystal structures are shown as black dots. Histograms attached to the graph show the distribution of χ_1 dihedral angles. See also Figures S2 and S3.

$sTPR_{L254F\text{-}C1}$ and $sTPR_{L254F\text{-}C1}$, $sTPR_{L254F}$ conformations 1 and 2; sTPR, simplified TPR; TPR, tetratricopeptide repeats.

up to 12 Å in the TPR helix, which ties in with the observed reduction in activity. Furthermore, using differential scanning fluorimetry and molecular dynamics simulations we show that the TPR helix is significantly destabilized, leading to defects in substrate recognition. This is a major advance for the *O*-GlcNAc field as it provides a molecular understanding of this mutation and provides a platform for exploring effects on the *O*-GlcNAc proteome.

STAR★METHODS

Detailed methods are provided in the online version of this paper and include the following:

- KEY RESOURCES TABLE
- CONTACT FOR REAGENT AND RESOURCE SHARING
- EXPERIMENTAL MODEL DETAILS
  - Cell Lines
- METHOD DETAILS
  - Molecular Cloning
  - OGT Expression and Purification
  - TPR Expression and Purification
  - TPR Crystallisation and Structural Analysis
  - Thermal Denaturing Assay
  - Molecular Dynamics Simulations
  - In Vitro O-GlcNAcylation Assays
- QUANTIFICATION AND STATISTICAL ANALYSIS
- DATA AND SOFTWARE AVAILABILITY

SUPPLEMENTAL INFORMATION

Supplemental Information includes three figures and can be found with this article online at https://doi.org/10.1016/j.chembiol.2018.03.004.

ACKNOWLEDGMENTS

This work funded by a Wellcome Trust Investigator award (110061) to D.M.F.v.A and a Wellcome Trust ISSF award (WT097818MF) to U.Z. M.G. was supported by a University of Dundee Translational Medical Research Fund Ph.D. fellowship. A.G. was supported by a Wellcome Trust PhD studentship. Diffraction experiments were performed on beamline ID30A-1 at the European Synchrotron Radiation Facility (ESRF), Grenoble, France. We are grateful to Matthew Bowler at the ESRF for providing assistance in using the beamline ID30A-1.

AUTHOR CONTRIBUTIONS

D.M.F.v.A. and U.Z. conceived the study; M.G. performed all protein production, *in vitro* assays, and protein crystallography experiments; M.G. and A.G. performed the OGT activity assay against HEK-293 cell lysate; S.L. performed molecular dynamics simulations; A.T.F. performed molecular biology experiments; M.G., A.G., and S.L. analyzed the data; M.G. and D.M.F.v.A. interpreted the data and wrote the manuscript with input from all authors.

DECLARATION OF INTERESTS

The authors declare no competing interests.

Received: November 27, 2017
Revised: January 30, 2018
Accepted: February 28, 2018
Published: March 29, 2018

REFERENCES

Abraham, M.J., Murtola, T., Schulz, R., Páall, S., Smith, J.C., Hess, B., and Lindah, E. (2015). Gromacs: high performance molecular simulations through multi-level parallelism from laptops to supercomputers. SoftwareX *1–2*, 19–25.

Battye, T.G., Kontogiannis, L., Johnson, O., Powell, H.R., and Leslie, A.G. (2011). iMOSFLM: a new graphical interface for diffraction-image processing with MOSFLM. Acta Crystallogr. D Biol. Crystallogr. *67*, 271–281.

Berendsen, H.J.C., Postma, J.P.M., van Gunsteren, W.F., DiNola, A., and Haak, J.R. (1984). Molecular dynamics with coupling to an external bath. J. Chem. Phys. *81*, 3684–3690.

Borodkin, V.S., Schimpl, M., Gundogdu, M., Rafie, K., Dorfmueller, H.C., Robinson, D.A., and van Aalten, D.M. (2014). Bisubstrate UDP-peptide conjugates as human O-GlcNAc transferase inhibitors. Biochem. J. *457*, 497–502.

Bouazzi, H., Lesca, G., Trujillo, C., Alwasiyah, M.K., and Munnich, A. (2015). Nonsyndromic X-linked intellectual deficiency in three brothers with a novel MED12 missense mutation [c.5922G>T (p.Glu1974His)]. Clin. Case Rep. *3*, 604–609.

Bussi, G., Donadio, D., and Parrinello, M. (2007). Canonical sampling through velocity rescaling. J. Chem. Phys. *126*, 014101.

Cole, R.N., and Hart, G.W. (2001). Cytosolic O-glycosylation is abundant in nerve terminals. J. Neurochem. *79*, 1080–1089.

Connarn, J.N., Assimon, V.A., Reed, R.A., Tse, E., Southworth, D.R., Zuiderweg, E.R., Gestwicki, J.E., and Sun, D. (2014). The molecular chaperone Hsp70 activates protein phosphatase 5 (PP5) by binding the tetratricopeptide repeat (TPR) domain. J. Biol. Chem. *289*, 2908–2917.

Darden, T., York, D., and Pedersen, L. (1993). Particle mesh Ewald: an N log(N) method for Ewald sums in large systems. J. Chem. Phys. *98*, 10089.

Deplus, R., Delatte, B., Schwinn, M.K., Defrance, M., Méndez, J., Murphy, N., Dawson, M.A., Volkmar, M., Putmans, P., Calonne, E., et al. (2013). TET2 and TET3 regulate GlcNAcylation and H3K4 methylation through OGT and SET1/COMPASS. EMBO J. *32*, 645–655.

Feenstra, K.A., Hess, B., and Berendsen, H.J.C. (1999). Improving efficiency of large time-scale molecular dynamics simulations of hydrogen-rich systems. J. Comput. Chem. *20*, 786–798.

Francisco, H., Kollins, K., Varghis, N., Vocadlo, D., Vosseller, K., and Gallo, G. (2009). O-GlcNAc post-translational modifications regulate the entry of neurons into an axon branching program. Dev. Neurobiol. *69*, 162–173.

Gorrec, F. (2015). The MORPHEUS II protein crystallization screen. Acta Crystallogr. F Struct. Biol. Commun. *71*, 831–837.

Gowers, R.J., Linke, M., Barnoud, J., Reddy, T.J.E., Melo, M.N., Seyler, S.L., Domański, J., Dotson, D.L., Buchoux, S., Kenney, I.M., et al. (2016). MDAnalysis: a Python package for the rapid analysis of molecular dynamics simulations. In: Proceedings of the 15th Python in Science Conference, pp. 98–105.

Hess, B., Bekker, H., Berendsen, H.J.C., and Fraaije, J.G.E.M. (1997). LINCS: a linear constraint solver for molecular simulations. J. Comput. Chem. *18*, 1463–1472.

Iyer, S.P., and Hart, G.W. (2003). Roles of the tetratricopeptide repeat domain in O-GlcNAc transferase targeting and protein substrate specificity. J. Biol. Chem. *278*, 24608–24616.

Jínek, M., Rehwinkel, J., Lazarus, B.D., Izaurralde, E., Hanover, J.A., and Conti, E. (2004). The superhelical TPR-repeat domain of O-linked GlcNAc transferase exhibits structural similarities to importin α. Nat. Struct. Mol. Biol. *11*, 1001–1007.

Joung, I.S., and Cheatham, T.E. (2008). Determination of alkali and halide monovalent ion parameters for use in explicitly solvated biomolecular simulations. J. Phys. Chem. B *112*, 9020–9041.

Kappel, C., Zachariae, U., Dölker, N., and Grubmüller, H. (2010). An unusual hydrophobic core confers extreme flexibility to HEAT repeat proteins. Biophys. J. *99*, 1596–1603.

Kochinke, K., Zweier, C., Nijhof, B., Fenckova, M., Cizek, P., Honti, F., Keerthikumar, S., Oortveld, M.A., Kleefstra, T., Kramer, J.M., et al. (2016). Systematic phenomics analysis deconvolutes genes mutated in intellectual disability into biologically coherent modules. Am. J. Hum. Genet. *98*, 149–164.

Lamb, J.R., Tugendreich, S., and Hieter, P. (1995). Tetratrico peptide repeat interactions: to TPR or not to TPR? Trends Biochem. Sci. *20*, 257–259.

Lazarus, M.B., Jiang, J., Kapuria, V., Bhuiyan, T., Janetzko, J., Zandberg, W.F., Vocadlo, D.J., Herr, W., and Walker, S. (2013). HCF-1 is cleaved in the active site of O-GlcNAc transferase. Science *342*, 1235–1239.

Lindorff-Larsen, K., Piana, S., Palmo, K., Maragakis, P., Klepeis, J.L., Dror, R.O., and Shaw, D.E. (2010). Improved side-chain torsion potentials for the Amber ff99SB protein force field. Proteins *78*, 1950–1958.

Lubas, W.A., Frank, D.W., Krause, M., and Hanover, J.A. (1997). O-linked GlcNAc transferase is a conserved nucleocytoplasmic protein containing tetratricopeptide repeats. J. Biol. Chem. *272*, 9316–9324.

März, P., Stetefeld, J., Bendfeldt, K., Nitsch, C., Reinstein, J., Shoeman, R.L., Dimitriades-Schmutz, B., Schwager, M., Leiser, D., Özcan, S., et al. (2006). Ataxin-10 interacts with O-linked β-N-acetylglucosamine transferase in the brain. J. Biol. Chem. *281*, 20263–20270.

McGibbon, R.T., Beauchamp, K.A., Harrigan, M.P., Klein, C., Swails, J.M., Hernandez, C.X., Schwantes, C.R., Wang, L.P., Lane, T.J., and Pande, V.S. (2015). MDTraj: a modern open library for the analysis of molecular dynamics trajectories. Biophys. J. *109*, 1528–1532.

Michaud-Agrawal, N., Denning, E.J., Woolf, T.B., and Beckstein, O. (2011). MDAnalysis: a toolkit for the analysis of molecular dynamics simulations. J. Comput. Chem. *32*, 2319–2327.

Miyamoto, S., and Kollman, P.A. (1992). SETTLE: an analytical version of the SHAKE and RATTLE algorithm for rigid water models. J. Comput. Chem. *13*, 952–962.

Niranjan, T.S., Skinner, C., May, M., Turner, T., Rose, R., Stevenson, R., Schwartz, C.E., and Wang, T. (2015). Affected kindred analysis of human X chromosome exomes to identify novel X-linked intellectual disability genes. PLoS One *10*, e0116454.

Okuyama, R., and Marshall, S. (2003). UDP-N-acetylglucosaminyl transferase (OGT) in brain tissue: temperature sensitivity and subcellular distribution of cytosolic and nuclear enzyme. J. Neurochem. *86*, 1271–1280.

Pathak, S., Alonso, J., Schimpl, M., Rafie, K., Blair, D.E., Borodkin, V.S., Schüttelkopf, A.W., Albarbarawi, O., and van Aalten, D.M.F. (2015). The active site of O-GlcNAc transferase imposes constraints on substrate sequence. Nat. Struct. Mol. Biol. *22*, 744–750.

Pekkurnaz, G., Trinidad, J.C., Wang, X., Kong, D., and Schwarz, T.L. (2014). Glucose regulates mitochondrial motility via Milton modification by O-GlcNAc transferase. Cell *158*, 54–68.

Rafie, K., Raimi, O., Ferenbach, A.T., Borodkin, V.S., Kapuria, V., and van Aalten, D.M.F. (2017). Recognition of a glycosylation substrate by the O-GlcNAc transferase TPR repeats. Open Biol. *7*, https://doi.org/10.1098/rsob.170078.

Ropers, H.H. (2010). Genetics of early onset cognitive impairment. Annu. Rev. Genomics Hum. Genet. *11*, 161–187.

Scheufler, C., Brinker, A., Bourenkov, G., Pegoraro, S., Moroder, L., Bartunik, H., Hartl, F.U., and Moarefi, I. (2000). Structure of TPR domain-peptide complexes: critical elements in the assembly of the Hsp70-Hsp90 multichaperone machine. Cell *101*, 199–210.

Timbers, T.A., Ardiel, E.L., Mcdiarmid, T.A., Lee, K.C.Y., Safaei, J., Pelech, S.L., and Rankin, C.H. (2017). CaMK (CMK-1) and O-GlcNAc transferase (OGT-1) modulate mechanosensory responding and habituation in an interstimulus interval-dependent manner in *Caenorhabditis elegans*. BioRxiv. https://doi.org/10.1101/115972.

Vagin, A.A., Steiner, R.A., Lebedev, A.A., Potterton, L., McNicholas, S., Long, F., and Murshudov, G.N. (2004). REFMAC5 dictionary: organization of prior

CellPress

chemical knowledge and guidelines for its use. Acta Crystallogr. D Biol. Crystallogr. *60*, 2184–2195.

Vaidyanathan, K., Niranjan, T., Selvan, N., Teo, C.F., May, M., Patel, S., Weatherly, B., Skinner, C., Opitz, J., Carey, J., et al. (2017). Identification and characterization of a missense mutation in the O-linked β-N-acetylglucosamine (O-GlcNAc) transferase gene that segregates with X-linked intellectual disability. J. Biol. Chem. *292*, 8948–8963.

Willems, A.P., Gundogdu, M., Kempers, M.J.E., Giltay, J.C., Pfundt, R., Elferink, M., Loza, B.F., Fuijkschot, J., Ferenbach, A.T., van Gassen, K.L.I., et al. (2017). Mutations in N-acetylglucosamine (O-GlcNAc) transferase in patients with X-linked intellectual disability. J. Biol. Chem. *292*, 12621–12631.

Winn, M.D., Ballard, C.C., Cowtan, K.D., Dodson, E.J., Emsley, P., Evans, P.R., Keegan, R.M., Krissinel, E.B., Leslie, A.G.W., McCoy, A., et al. (2011). Overview of the CCP4 suite and current developments. Acta Crystallogr. D Biol. Crystallogr. *67*, 235–242.

Yang, X., and Qian, K. (2017). Protein O-GlcNAcylation: emerging mechanisms and functions. Nat. Rev. Mol. Cell Biol. *18*, 452–465.

Yang, X., Zhang, F., and Kudlow, J.E. (2002). Recruitment of O-GlcNAc transferase to promoters by corepressor mSin3A: coupling protein O-GlcNAcylation to transcriptional repression. Cell *110*, 69–80.

Zeytuni, N., and Zarivach, R. (2012). Structural and functional discussion of the tetra-trico-peptide repeat, a protein interaction module. Structure *20*, 397–405.

STAR★METHODS

KEY RESOURCES TABLE

REAGENT or RESOURCE	SOURCE	IDENTIFIER
Antibodies		
Mouse monoclonal anti *O*-GlcNAc (RL2)	Abcam	ab2739; RRID:AB_303264
Rabbit polyclonal anti HSP90	Cell Signalling	Cat#4874; RRID:AB_2121214
Rabbit polyclonal anti HSP90	Enzo Life Sciences	ADI-SPA-836-D; RRID:AB_991589
Rabbit polyclonal anti OGT (H-300)	Santa Cruz Biotechnology	sc-32921
Goat IRDye® 680RD anti-Mouse	LICOR	P/N 925-68070; RRID:AB_621840
Donkey IRDye® 800CW anti-Rabbit	LICOR	P/N 925-32213; RRID:AB_621848
Morpheus® HT-96 Crystal Screen	Molecular Dimensions	MD1-47
Bacterial and Virus Strains		
E. coli BL21-DE3	New England Biotechnologies	C25271
Chemicals, Peptides, and Recombinant Proteins		
SYPRO® Orange dye	Sigma	S5692-50UL
GlcNAcstatin-G (OGA inhibitor)	GlycoBioChem	GBC10002
RB2 (residues 410-422)	GlycoBioChem	N/A
*Cp*OGA	GlycoBioChem	N/A
$OGT_{WT/L254F}$	This paper	N/A
$TPR_{WT/L254F}$	This paper	N/A
Deposited Data		
TPR_{L254F} structure	This paper	PDB: 6EOU
Experimental Models: Cell Lines		
HEK-293	ATCC	CRL-1573
Oligonucleotides		
Primer TPR: Forward CTGGGATCCGGCCCGATGGAACT GGGCTCATCGTGAAATATCAG	This paper	N/A
Primer TPR: Reverse GATGCGGCCGCTTAGTCTTGCATT TCTTTCAGCGTATTAC	This paper	N/A
Recombinant DNA		
pGEX-6P-1 Vector	GE Healthcare	28954648
pHEX-6P-1 Vector	This paper	N/A
Software and Algorithms		
Graphpad Prism v5.0	GraphPad Software	https://www.graphpad.com/
iMosflm	(Battye et al., 2011)	http://www.ccp4.ac.uk/
CCP4	(Winn et al., 2011)	http://www.ccp4.ac.uk/
REFMAC	(Vagin et al., 2004),	http://www.ccp4.ac.uk/
GROMACS	(Abraham et al., 2015).	http://www.gromacs.org/
MDAnalysis	(Michaud-Agrawal et al., 2011)	https://www.mdanalysis.org/
MDTraj	(McGibbon et al., 2015)	http://mdtraj.org/1.9.0/
Other		
Morpheus® HT-96 Crystal Screen	Molecular Dimensions	MD1-47

CONTACT FOR REAGENT AND RESOURCE SHARING

Further information and requests for resources and reagents should be directed to and will be fulfilled by the lead contact, Daan M. F. van Aalten (dmfvanaalten@dundee.ac.uk).

EXPERIMENTAL MODEL DETAILS

Cell Lines

Female HEK-293 cells were obtained from ATCC. Due to the use of HEK-293 lysates solely for biochemistry, the cell line was not further authenticated. The cells were tested negative for mycoplasma contamination (October 2017). HEK-293 cells were grown on 15 cm plates in DMEM (Life Technologies) supplemented with 2 mM L-glutamine (Sigma), 100 units/ml Penicillin and 100 μg/ml Streptomycin (Life Technologies), and 10% foetal calf serum (Labtech).

METHOD DETAILS

Molecular Cloning

The full-length codon optimised OGT was obtained from GenScript and subcloned as a *Bam*HI-*Not*I fragment into pHEX-6P-1 (modified version of pGEX-6P-1 which contains a 6His tag instead of GST). The L254F mutation was introduced using a method similar to the QuikChange site-directed mutagenesis kit by Agilent but using KOD polymerase and *Dpn*I from Fermentas. All inserts were confirmed by DNA sequencing. The TPR region of OGT (residues 26-410) was amplified from both the OGT_{WT} and OGT_{L254F} expression constructs. These were cloned into pGEX-6P-1 as *Bam*HI-*Not*I fragments and the inserts were confirmed by DNA sequencing. Forward and reverse primers used were CTGGGATCCGGCCCGATGGAACTGGGCTCATC-GTGAAATATCAG and GATGCGGC CGCTTAGTCTTGCATTTCTTTCAGCGTATTAC, respectively.

OGT Expression and Purification

Full length OGT_{WT} and OGT_{L254F} were expressed in *E. coli* BL21-DE3 as N-terminal His fusion proteins as described previously (Willems et al., 2017). Briefly, transformed *E. coli* cells were grown in autoinduction medium at 37°C with agitation until OD_{600} reached 0.8, at which point the temperature was lowered to 18°C for overnight incubation. Cells were harvested by centrifugation at 4°C (35 min 4,500 × *g*). Resulting cell pellet was resuspended in base buffer (0.1 M Tris-HCl, pH 7.5, 0.15 M NaCl, 0.5 mM TCEP (tris[2-carboxyethyl]phosphine) supplemented with 25 mM imidazole 0.1 mg/ml DNase I and protease inhibitor cocktail (1 mM benzamidine, 0.2 mM PMSF, 5 mM leupeptin), and lysed via continuous flow cell disruptor (three passes at 15,000 PSI). Lysate was clarified by centrifugation (30,000 g for 1 h at 4°C) followed by incubation with 1 ml per litre of culture of Ni^{2+}-NTA agarose resin (GE Healthcare) for 2 h at 4°C. The resin was thoroughly washed and eluted with base buffer supplemented with 25 mM and 500 mM imidazole respectively. Eluted protein was dialyzed and cleaved from the His-tag overnight at 4°C in buffer A (0.1 M tris-HCl, pH 8.5, 25 mM NaCl) supplemented with PreScission protease (GE Healthcare), then passed through fresh Ni^{2+}-NTA agarose resin. Dialyzed protein was loaded onto 5 ml HiTrap Q Sepharose FF anion exchange resin (GE Healthcare) and eluted with a linear gradient up to 60% of buffer B (0.1 M tris-HCl, pH 8.5, 500 mM NaCl). Peak fractions were pooled, concentrated and further purified via size exclusion chromatography using 300-ml prepacked Superdex™ 200 column (GE Healthcare) equilibrated with base buffer. The peak fractions were concentrated to 10 mg/ml, mixed 1:1 with 50% glycerol, snap-frozen and stored at -80°C until use.

TPR Expression and Purification

The TPR region of OGT (residues 26-410), either wild type (TPR_{WT}) or bearing the L254F mutation (TPR_{L254F}), was expressed and purified as N-terminally GST fusion proteins as described previously (Jínek et al., 2004). Briefly, *E. coli* BL21-DE3 cells were transformed, grown and harvested as described for the full length OGT constructs. Resulting cell pellet was resuspended in base buffer (20 mM Na-HEPES, pH 7.5, 200 mM NaCl, 2 mM DTT supplemented with 0.1 mg/ml DNase I and protease inhibitor cocktail (1 mM benzamidine, 0.2 mM PMSF, 5 mM leupeptin), prior to being lysed and clarified as described above. Clarified lysate was then incubation with 1 ml per litre of culture of Glutathione Sepharose 4B resin (GE Healthcare) for 2 h at 4°C. The resin was thoroughly washed with base and the recombinant proteins were cleaved on-resin by addition of PreScission protease (GE Healthcare) and overnight incubation at 4°C. Cleaved protein was eluted, concentrated and further purified via size exclusion chromatography using 300-ml prepacked Superdex™ 200 column (GE Healthcare) equilibrated with base buffer. The peak fractions were concentrated to 30 mg/ml, snap-frozen in liquid nitrogen and stored at -80°C until use.

TPR Crystallisation and Structural Analysis

Crystallisation of TPR_{L254F} was performed at 22°C using MRC 96-well sitting drop crystallization plates (Molecular Dimensions) by combining 0.2 μl TPR_{L254F} (in 20 mM Na-HEPES-NaOH pH 7.5, 200 mM NaCl and 2 mM DTT) with 0.2 μl of reservoir solution (0.1 M Na-HEPES and 0.1 M MOPS-HCl pH 7,5, 0.04 M diethylene glycol, 0.04 M triethylene glycol, 0.04 M tetraethylene glycol, 0.04 M pentaethylene glycol, 20 % v/v ethylene glycol and 10 % w/v PEG 4000) (Morpheus®, Molecular Dimensions (Gorrec, 2015)). Orthorhombic rod and disc shaped crystals appeared within 1-2 days. Prior to diffraction experiments, individual crystals were flash-frozen in liquid nitrogen without prior cryoprotection. Diffraction data were collected at the European Synchrotron Radiation Facility beamline ID30A-1. Data were processed with iMosflm (Battye et al., 2011) and scaled to 1.75 Å using SCALA (Winn et al., 2011). The structure was solved by molecular replacement using the structure for TPR_{WT} (PDB: 1W3B Jínek et al., 2004) as the search model. The resulting model was initially truncated at both N- and C-termini where the fit of the electron density and the model was poor, and manually rebuilt and refined using Coot (Winn et al., 2011) and REFMAC (Vagin et al., 2004), respectively. The editing and

refinement of the model was iterated until it was in complete agreement with the data. Scaling and model building statistics can be seen in Table 1.

Thermal Denaturing Assay

Thermal denaturation experiments were performed in triplicate, using constructs encompassing the TPR domain (residues 26-410). 50 μl solutions contained 5 μM protein and 1.1x SYPRO® Orange dye (Sigma) in base buffer of 25 mM HEPES-NaOH pH 7.5, 150 mM NaCl and 0.5 mM TCEP. CFX Connect™ Real-Time System (BIO-RAD) was used to measure fluorescence (λ_{ex} = 530 nm, λ_{em} = 560 nm) while temperature was increased from 25 to 95°C at 1 degree per minute increments. The data were transformed, normalised and fitted to a four-parameter Boltzmann sigmoidal curve using GraphPad Prism 5.0.

Molecular Dynamics Simulations

Truncated OGT TPR wild type and L254F constructs comprising TPRs 6-8 ($sTPR_{WT}$ and $sTPR_{L254F}$; residues 189-294, Figure 1A) were used in molecular dynamics simulations, similar to an approach previously used to simulate sections of the alpha-solenoid HEAT repeat protein importin-β (Kappel et al., 2010). Appropriate capping groups were added to N- and C-terminal ends of both $sTPR_{WT}$ and $sTPR_{L254F}$ constructs. The major axes of the sTPR constructs were aligned to the z-axis of a triclinic simulation box a triclinic box of 62.5 x 62.5 x 82.5 Å and solvated using explicit water molecules. Na^+ and Cl^- ions were added in order to neutralise the system at the physiological NaCl concentration of 0.15 mM. The amber99SB-ildn force field (Lindorff-Larsen et al., 2010) and virtual sites for hydrogen atoms (Feenstra et al., 1999) were used. The TIP3P water model was used to model the solvent molecules and Joung and Cheatham III parameters (Joung and Cheatham, 2008) were used to model the counter ions. Simulations were carried out with the GROMACS molecular dynamics package, version 5.1.5 (Abraham et al., 2015). For each system, the geometry was minimized in four cycles that combined 3500 steps of steepest descent algorithm followed by 4500 of conjugate gradient. Thermalisation of the system was performed in 6 steps of 5 ns, where the temperature was gradually increased from 50 K to 298 K, while the protein was restrained with a force constant of 10 kJ mol^{-1} $Å^{-2}$. Production runs consisted of four replicates of 500 ns simulations for each system (accounting for 2 μs of simulation time per system). Making use of virtual sites, the integration time-step was set to 4 fs. Temperature was kept constant by weakly coupling (t = 0.1 ps) protein and water and ions separately to a temperature bath of 298K with the velocity rescale thermostat of Bussi et al. (Bussi et al., 2007). The pressure was kept constant at 1 bar using semi-isotropic Berendsen coupling (Berendsen et al., 1984). Long-range electrostatic interactions were calculated using the smooth particle mesh Ewald method (Darden et al., 1993) beyond a short-range Coulomb cut-off of 10 Å. A 10-Å cut-off was also set for Lennard-Jones interactions. The LINCS algorithm (Hess et al., 1997) was used to restrain the bonds involving hydrogen and the SETTLE algorithm (Miyamoto and Kollman, 1992) was used to constrain bond lengths and angles of water molecules. Periodic boundary conditions were applied.

In Vitro O-GlcNAcylation Assays

Michaelis-Menten kinetics of OGT were measured using a fluorimetric assay as described previously (Borodkin et al., 2014), with the exception of reduced reaction volume of 25 μl and usage of 384-well plate. As acceptor substrate, a 13 amino acid long sequence from retinoblastoma-like protein 2 (RB2; $_{410}$KENSPAVTPVSTA$_{422}$; GlycoBioChem) was used. Reactions for Michaelis-Menten kinetics contained 0-768 μM acceptor peptide substrate, 200 μM UDP-GlcNAc and 50 nM OGT in 50 mM HEPES-NaOH pH 7.5, 0.1 mg/ml BSA and 10 μM $Na_2S_2O_4$. Reactions were stopped before 10% of the acceptor substrate was depleted by addition of 50 μl detection reagent (25 mM HEPES-NaOH pH 7.5, 10 mM NaCl, 15 μM xanthene based Zn(II) complex, 75 μM pyrocathecol violet and 50% methanol). The fluorescence was read using excitation and emission wavelengths of 485 nm and 530 nm, respectively. Data were background corrected and plotted using GraphPad Prism.

Additional *O*-GlcNAcylation assays were performed on de-*O*-GlcNAcylated HEK-293 lysate proteins. Cultured HEK-293 cells were washed twice with ice-cold PBS buffer (Life Technologies) prior to lysis. Cells were lysed by addition of lysis buffer (50 mM Tris-HCl, pH 7.4, 1 mM EGTA, 1 mM EDTA, 1% Triton-X100, 1 mM Na_3VO_4, 50 mM NaF, 5 mM $Na_4P_2O_7$, 0.27 M sucrose) supplemented with 1 μM ß-mercaptoethanol, 1 mM benzamidine, 0.2 mM PMSF and 5 mM leupeptin. The lysate was transferred into microfuge tubes and clarified by centrifugation at 4°C (17,000 g for 15 min). The lysate was then treated with 120 μg *Cp*OGA per mg of lysate protein and incubated for 90 minutes at 37°C. *Cp*OGA and endogenous HEK-293 OGA were then neutralised by addition of 250 μM GlcNAcstatin-G, an OGA inhibitor. Reactions were then supplemented with OGT_{WT} or OGT_{L254F} (0.2 μM) in presence of 2 mM UDP-GlcNAc and incubated for an additional 2 h at 37°C. Proteins were resolved by SDS-PAGE (3-8% Tris-Acetate gels; Life Technologies), transferred onto nitrocellulose membrane (GE Healthcare), and probed using *O*-GlcNAc-RL2 (1:1,000 dilution; Abcam), HSP90 (1:5,000 dilution; Cell Signalling or Enzo Life Sciences) and OGT-H300 (1:1,000 dilution; Santa Cruz) primary antibodies and corresponding IRDye associated secondary antibodies (1:10,000 dilution; LI-COR). Resulting signal was quantified using a LI-COR Odyssey scanner and associated quantification software. Data were plotted using GraphPad Prism 5.0.

QUANTIFICATION AND STATISTICAL ANALYSIS

Michaelis-Menten kinetics of the OGT-catalysed reaction against the peptide substrate derived from RB2 was performed as three technical replicates and repeated two times (data presented in Figure S1A). The *O*-GlcNAc activity assay against deglycosylated HEK-293 cell lysate proteins was repeated six times (data presented in Figures 1B and S1B). The ThermoFluor assay was performed

as seven technical replicates, and repeated two times (data presented in Figure 2A). For all activity and thermal stability assays, GraphPad Prism was used for calculation of statistics. Error bars represent the standard deviation of the mean in all presented data. The trajectories obtained by the molecular dynamics simulations were analysed with the MDAnalysis (RMSD and RMSF) (Gowers et al., 2016; Michaud-Agrawal et al., 2011) and MDtraj (distances and angles) (McGibbon et al., 2015) packages. The values reported in the results and discussion sections correspond to the mean values ± standard deviation of the mean.

DATA AND SOFTWARE AVAILABILITY

The crystallographic structure has been deposited in RCSB Protein Data Bank (http://www.rcsb.org/pdb) under ID code 6EOU.

Cell Chemical Biology, Volume *25*

Supplemental Information

The *O*-GlcNAc Transferase Intellectual Disability Mutation L254F Distorts the TPR Helix

Mehmet Gundogdu, Salomé Llabrés, Andrii Gorelik, Andrew T. Ferenbach, Ulrich Zachariae, and Daan M.F. van Aalten

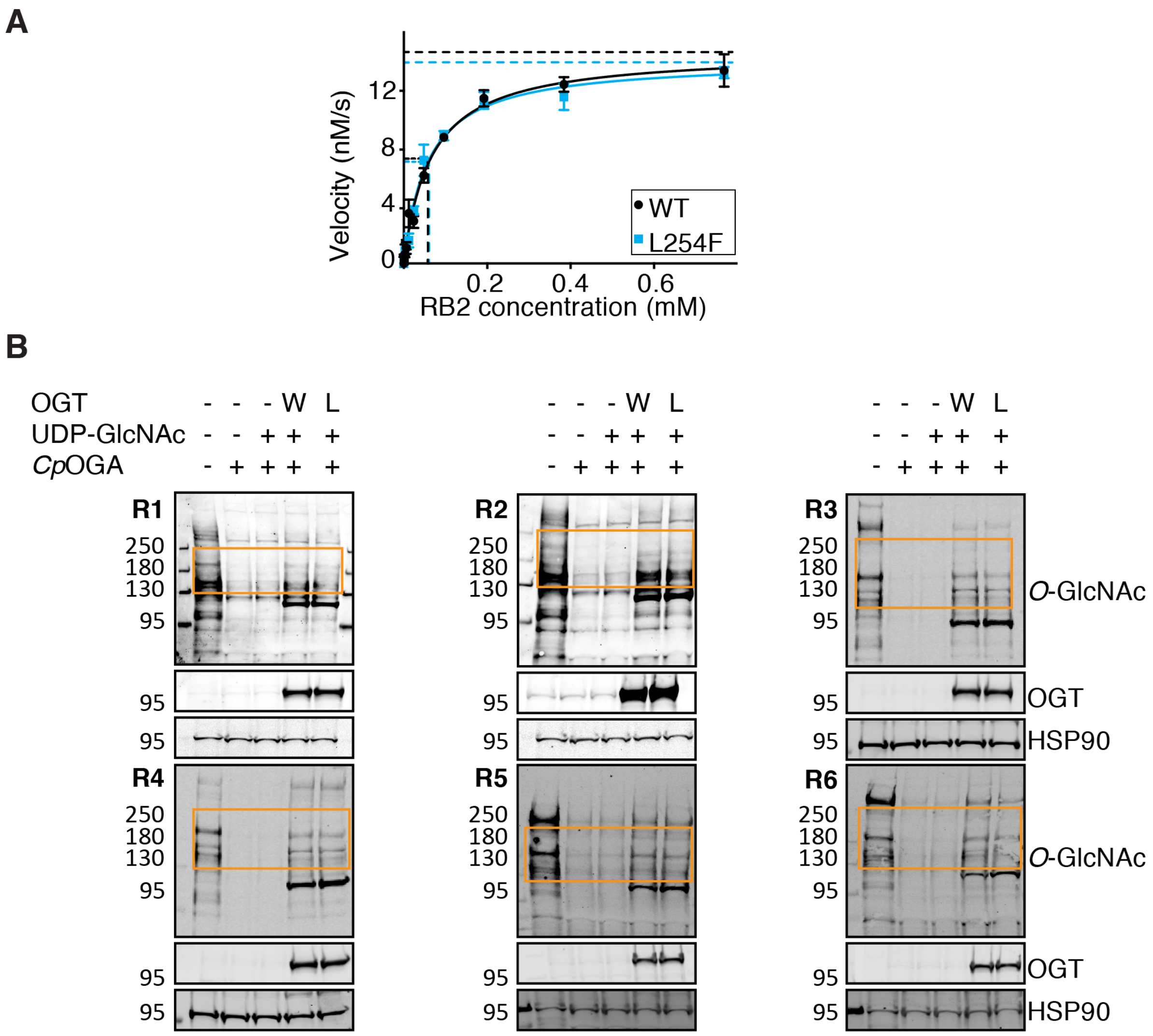

Supplemental figure 1: *in vitro* characterisation of OGT$_{L254F}$. Related to Figure 1B

(A) Michaelis-Menten kinetics of OGT wild type and mutant against RB2 peptide. X- and y-axis intercepts of the dashed lines mark the Michaelis constant (K_M) and maximum velocity (V_{max}) of the reaction. Error bars represent standard deviation of three replicates. **(B)** Western blot analyses showing OGT activity against de-*O*-GlcNAcylated HEK-293 cell lysate. Results represent six biological replicates and orange boxes depict the area that was used for quantification. *Cp*OGA: *Clostridium perfringens*; L: L254F; W: wild type.

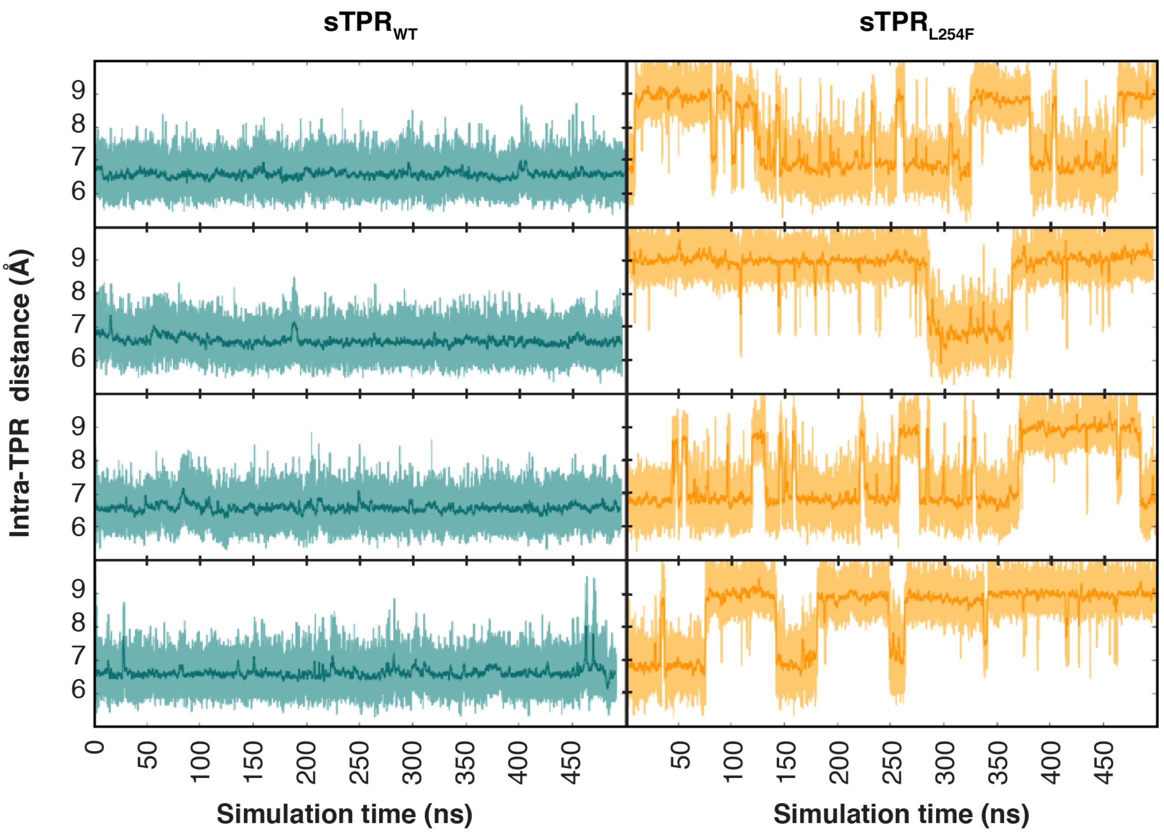

Supplemental figure 2: Analysis of the intra-TPR repeat distance. Related to Figures 2B and 2C

Molecular dynamics simulation data investigating the intra TPR distance observed in $sTPR_{WT}$ and $sTPR_{L254F}$ are shown on left and right panels, respectively. sTPR: simplified TPR; TPR: tetratricopeptide repeat.

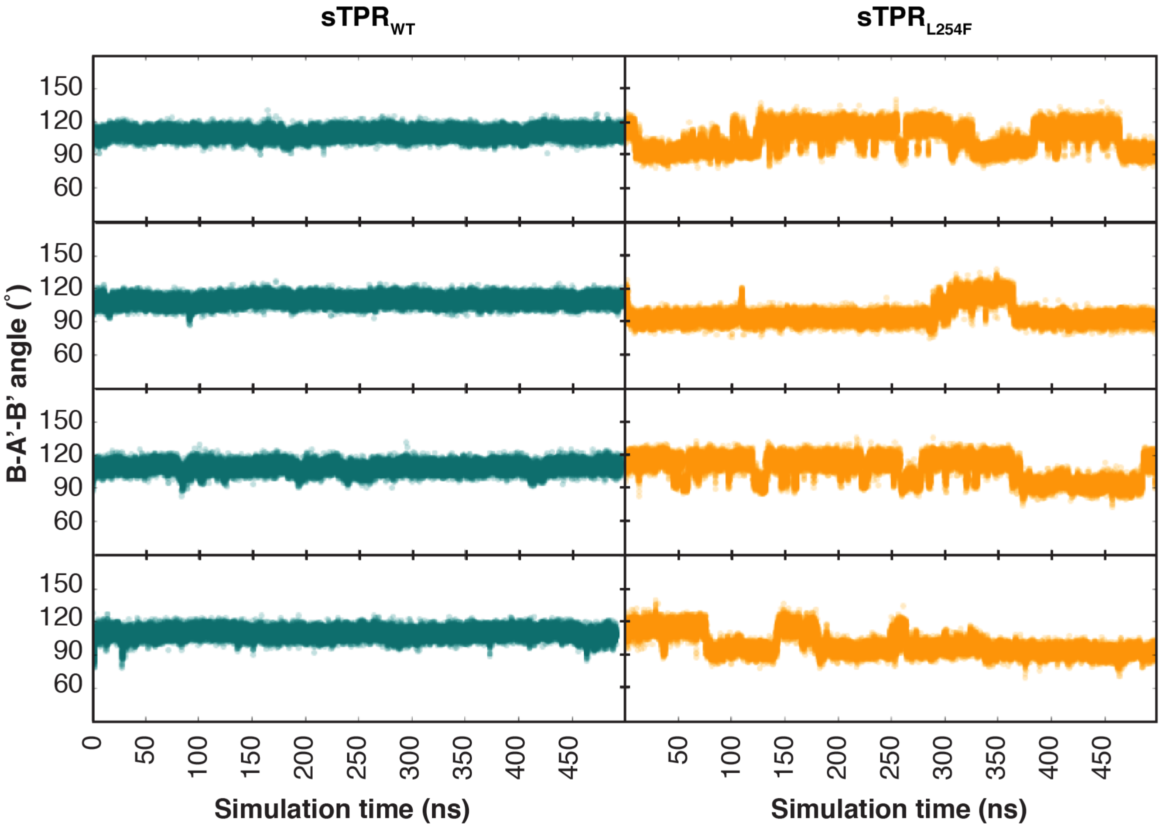

Supplemental figure 3: Analysis of the angle B-A'-B'. Related to Figures 2B and 2C

Molecular dynamics simulation data investigating the B-A'-B' angle observed in $sTPR_{WT}$ and $sTPR_{L254F}$ are shown on left and right panels, respectively. sTPR: simplified TPR; TPR: tetratricopeptide repeat.
